# Chemical Profile and Evaluation of the Antioxidant, Anti-Enzymatic, and Antibacterial Activity of *Astragalus strictispinus* and *Astragalus macrocephalus* subsp. *finitimus*

**DOI:** 10.3390/plants14223485

**Published:** 2025-11-15

**Authors:** Saba Shahrivari-Baviloliaei, Ilkay Erdogan Orhan, Fatma Sezer Senol Deniz, Mustafa Abdullah Yilmaz, Agnieszka Viapiana, Agnieszka Konopacka, Osman Tugay, Alina Plenis

**Affiliations:** 1 Department of Analytical Chemistry, Faculty of Pharmacy, Medical University of Gdansk, 80-210 Gdansk, Poland; agnieszka.viapiana@gumed.edu.pl (A.V.); alina.plenis@gumed.edu.pl (A.P.); 2 Department of Pharmacognosy, Faculty of Pharmacy, Gazi University, 06330 Ankara, Türkiye; 3 Department of Pharmacognosy, Faculty of Pharmacy, Lokman Hekim University, 06510 Ankara, Türkiye; 4 Department of Analytical Chemistry, Faculty of Pharmacy, Dicle University, 21280 Diyarbakır, Türkiye; 5 Department of Pharmaceutical Microbiology, Faculty of Pharmacy, Medical University of Gdansk, 80-210 Gdansk, Poland; 6 Department of Pharmaceutical Botany, Faculty of Pharmacy, Selcuk University, 42130 Konya, Türkiye; otugay@selcuk.edu.tr

**Keywords:** *Astragalus*, antioxidant activity, anti-enzymatic activity, antibacterial activity, chemical composition

## Abstract

*Astragalus* species are characterized by rich active compounds, mainly polysaccharides, saponins, and polyphenols, with various important bioactivities, such as antioxidant, antitumor, anti-diabetes, antiviral, etc. In this study, the chemical profiles of ethanol, ethyl acetate, and dichloromethane extracts from different parts (leaves, flowers, and roots) of two endemic *Astragalus* species growing in Türkiye, i.e., *A. strictispinus* and *A. macrocephalus* subsp. *finitimus* were determined, along with their antioxidant, anti-enzymatic, and antibacterial properties. According to the results, naringenin and apigenin were identified as two common phenolic compounds of both *Astragalus* species, while only ethanol extracts of the roots and leaves and ethyl acetate extracts of flowers of *A. strictispinis* exhibited a low level of antioxidant activity (5–16%). Moreover, AChE and BChE inhibitory activities were higher in the ethyl acetate extract of *A. macrocephalus* subsp. *finitimus* leaves, while all leaf extracts of the analyzed *Astragalus* species, except dichloromethane extract of *A. strictispinus*, exhibited antibacterial activity against *S. aureus*. In conclusion, this study provides detailed information that may serve as the scientific basis for the use of *Astragalus* species as sources of bioactive compounds with multiple functions in the nutraceutical, cosmetic, and pharmaceutical industries.

## 1. Introduction

The Fabaceae family has more than 700 genera, of which *Astragalus* is the largest one. This genus has about 2900 species. Iran, with about 750 species, is one of the diversity centers of this genus, and there are approximately 485 species of *Astragalus* in Türkiye [1,2]. Some species, such as *Astragalus membranaceus* Fisch. Ex Bunge, are widely used in folk medicine, including traditional Chinese medicine. *Astragalus* species are used to treat problems such as inflammation, infection, fatigue, diarrhea, hepatoprotective, sore throats, diabetes, leukemia, toothaches, etc. [3,4]. They contain biologically active compounds such as flavonoids (apigenin, kaempferol, naringenin) [5], saponins (astragalosides) [6], and polysaccharides (*Astragalus* polysaccharide) [7], which exhibit various pharmacological activities and demonstrate antioxidant, anti-inflammatory, and antimicrobial properties. It has been demonstrated that *Astragalus* polysaccharides (APS), the most important natural active component, stimulate macrophage functions and regulate the expression of cytokines such as interleukin (IL)-1 and IL-6, as well as the production of nitric oxide [8]. Macrophages play a central role in the immune/inflammatory response through the production of several pro-inflammatory mediators, such as arachidonic acid metabolites, nitric oxide, and cytokines. These mediators are responsible for the typical hallmarks of inflammation. In addition, APS reduces the levels of blood glucose, increases the sensitivity to insulin, improves insulin resistance, and inhibits the apoptosis of islet β cells [9]. It also plays a key role in the treatment of diabetes mellitus and its complications. It was found that APS also exerted a certain bacteriostatic effect on the main pathogenic bacteria causing mastitis in dairy cattle, including *Streptococcus*, *Escherichia coli*, and *Staphylococcus aureus*; this bacteriostatic effect was dose-dependent [10]. At concentrations of 20 mg/L and 40 mg/L, APS also significantly inhibited the bacterial strains of *Staphylococcus aureus*, *Escherichia coli*, and *Salmonella* in vitro [11]. *Astragalus membranaceus* exerts its antioxidant effects by increasing the activity of endogenous antioxidant enzymes like superoxide dismutase (SOD), decreasing oxidative stress markers such as malondialdehyde (MDA), and scavenging free radicals directly. It provides significant protection against heart, brain, kidney, intestine, liver, and lung injury in various models of oxidative stress-related disease [12]. The bioactive compounds in the genus *Astragalus* offer significant potential for developing new therapeutic methods, and this fact makes them an attractive subject for researchers [13].

Natural products provide effective and safer alternatives for treating diseases [14]. In the case of *Astragalus*, its low side effect profile and high therapeutic efficacy make it prominent in alternative medicine. Moreover, *Astragalus*, when used in combination with treatments like chemotherapy and radiotherapy, enhances treatment efficacy and reduces toxicity [15]. Cheng et al. [16] evaluate the efficacy and safety of *Astragalus*-containing traditional Chinese medicine combined with platinum-based chemotherapy (PBC) in patients with advanced gastric cancer. The patients were given *Astragalus*-based herbal therapy combined with PBC. Any form of *Astragalus* preparation, including water decoction, extracts, granules, or injection, among other forms, regardless of administration route. The obtained results showed that *Astragalus*-containing traditional Chinese medicine combined with PBC had better efficacy and fewer side effects in the treatment of advanced gastric cancer. Cheng et al. [17] evaluated the therapeutic effects and mechanisms of *A. membranaceus* stems and leaves in alleviating memory impairment, as well as to identify its active ingredients responsible for such effects. Using a mouse model of memory deficits induced by D-gal combined with AlCl_3_, it was demonstrated that this plant significantly alleviated memory impairment. Cheng et al. [16] investigated the anti-inflammatory effects of *A. mongholicus* Bunge water extract (AWE). An imiquimod (IMQ)-induced psoriasis-like skin inflammation mouse model was used for investigating anti-psoriatic effects. The obtained results showed that AWE exhibited anti-oxidation and anti-inflammatory properties, while in mice with psoriasis-like skin inflammation, the administration of topical AWE reduced both the affected area and the severity index score. In addition, AWE exhibited direct anti-inflammatory effects by inhibiting neutrophil activation and anti-psoriatic effects in mice with IMQ-induced psoriasis-like skin inflammation.

Nowadays, plants with anticholinesterase effects are studied because of their potential role in contributing to Alzheimer’s (AD) and Parkinson’s disease. Acetylcholine is the neurotransmitter at synapses and within the central nervous system, while the reduction of acetylcholine through hydrolyzation by acetylcholinesterase (AChE) is the main cause of AD [18]. Butyrylcholinesterase (BChE), as the sister enzyme of AChE, also has a similar effect on butyrylcholine in dementia. Cholinergic deficiency is a hallmark of several cognitive disorders, making cholinesterase inhibitors a cornerstone of therapy, as they work by increasing acetylcholine levels in the brain to improve synaptic transmission [19]. Cholinesterase inhibitors function by preventing the breakdown of the neurotransmitter acetylcholine (ACh) in the brain and nervous system. Butyrylcholinesterase (BChE) is an enzyme that, like acetylcholinesterase (AChE), breaks down acetylcholine. However, its primary function and role in the body are more complex and less understood than those of AChE. In the later stages of diseases like Alzheimer’s, AChE activity declines, and BChE may take on a more significant role in regulating acetylcholine levels. By inhibiting BChE, more acetylcholine is available to support cognitive function [20]. While AChE is a primary target in the early stages of Alzheimer’s, BChE is considered a viable target for treatment, especially as the disease progresses and its role in neurotransmission becomes more prominent. Therefore, anticholinesterase drugs are prescribed widely for the treatment of AD. On the other hand, oxidative damage in neurons is another factor in the pathophysiology of AD. Hence, more ideal drug candidates showing both cholinesterase inhibition and antioxidant activity are being searched.

The genus name *Astragalus* is derived from the Greek word “astragalos”, meaning “heel bone,” and is thought to refer to the shape of its seeds. *Astragalus strictispinis* Boiss. is one of the endemic plants of Türkiye with not enough data about its biological activities. Cyclooctane triterpenoids, such as undescribed cycloartanes, flavonoids, lignans, steroidal glycosides, etc., were identified and isolated in the ethanol extract of *A. strictispinis* roots, which were collected from Kumalar Mountain (Afyonkarahisar, Türkiye). The methanol extract of the roots was reported to contain triterpenoid saponins, such as cycloastragenol, cycloasalgenin, 17-epicycloasalgenin, cycloastragenol-6-O-β-D-glucopyranoside, cycloastragenol-16-O-β-D-glucopyranoside, and cycloasgenin-6-O-β-D-glucopyranoside [21]. Cycloasalgenin is a triterpenoid compound with anti-inflammatory potential that is found in the genus *Astragalus*, while cycloastragenol is a triterpenoid compound found for the first time in *A. membranaceus* with anti-inflammatory and anti-aging effects [22]. Chen et al. [23] investigated the ameliorative functions of cycloastragenol in cecal ligation and puncture (CLP)-induced systemic inflammation in sepsis and lipopolysaccharide (LPS)-mediated inflammatory response and the impact of a Toll-like receptor 4 (TLR4) pathway on the anti-inflammatory effects of cycloastragenol. The results showed that cycloastragenol inhibits inflammatory factor production within RAW264.7 and THP-1 cells after LPS stimulation through suppressing the TLR4/MAPK/NF-κB pathway. In another study, Bagalagel et al. [24] evaluated the potential therapeutic effects of cycloastragenol in experimentally induced ulcerative colitis rats and examined the modulation of sphingosine kinase (SphK), macrophage inflammatory protein (MIP)-1α, and miR-143. Ulcerative colitis rats were treated with 30 mg/kg cycloastragenol, and the gene and protein expression levels of SphK, MIP-1α, B-cell lymphoma 2 (BCL2), BCL2-associated X (BAX), miR-143, NF-κB, tumor necrosis factor (TNF)-α, and active caspase-3 were assessed. The results showed that cycloastragenol treatment improved the induced morphological changes, and in ulcerative colitis rats, this compound significantly reduced expression levels of SphK, MIP-1α, BAX, NF-κB, TNF-α, and active caspase-3, associated with BCL2 and miR-143 overexpression. *Astragalus macrocephalus* Willd. subsp. *finitimus* (Bunge) D.F.Chamb. is native to Iran, Türkiye, Lebanon, Syria, and Transcaucasia. The methanol extract of different parts of this plant from Türkiye had apigenin, hyperoside, ferulic, and *p*-coumaric acids as the main compounds [25]. Moreover, the antioxidant activity of its methanol extract was evaluated by 2,2-diphenyl-1-picrylhydrazyl (DPPH), ferric-reducing antioxidant power (FRAP), cupric-reducing antioxidant capacity (CUPRAC), and 2,2′-azino-bis(3-ethylbenzothiazoline-6-sulfonic acid (ABTS) assays, and inhibitory effects of α-amylase and tyrosinase were investigated [25].

To the best of our knowledge, only a scarce amount of information is available on *A. strictispinus* and *A. macrocephalus* subsp. *finitimus*. *A. strictispinus* is an endemic and poorly studied species, whereas *A. macrocephalus* subsp. *finitimus* is more widespread but only partially investigated. Therefore, the current study aims to represent an intriguing opportunity to elucidate chemical composition, antioxidant, anticholinesterase, and antibacterial activities of the two mentioned *Astragalus* species growing in a specific geographical region and obtain detailed insights into chemical properties to address significant gaps in the literature. In addition, we hypothesized that differences in phytochemical composition between these two species would be translated into distinct enzymatic inhibitory and antibacterial activities. To the best of our knowledge, this is the first report on the enzyme inhibitory, antibacterial activity, DMPD radical scavenging, and PRAP activity of *A. strictispinus* and *A. macrocephalus* subsp. *finitimus*. The use of effective solvents, such as ethanol, ethyl acetate, and dichloromethane, ensured reliable and reproducible results. This is the first comprehensive study on anticholinesterase and the antibacterial activity of these *Astragalus* species.

## 2. Results and Discussion

### 2.1. Phenolic Compounds in the Extracts of the Astragalus Species

This is the first report of the quantification of bioactive compounds in the extracts of Turkish *A. macrocephalus* subsp. *finitimus* and *A. strictispinis*. In the current study, eighteen compounds (Table 1) were identified as cycloalkane (1), stilbenoid (23), hydroxycarboxylic acid (29), flavonoids (30, 33, 34, 35, 40, 42, 44, 48, 50, 51, 53, 54, and 56), and phenolic acids (6 and 17). In all extracts of *A. macrocephalus* subsp. *finitimus* and *A. strictispinis*, flavonoids, especially naringenin, luteolin, and apigenin, were the most common compounds, while in the phenolic acid group, protocatechuic acid was found in higher amounts than caffeic acid. Generally, the ethanol extracts contained a higher content of bioactive compounds than other extracts. Among the detected compounds in ethanolic extracts, the content of isoquercetin was the highest (above 1 mg/g dw extract) in the *A. macrocephalus* subsp. *finitimus* leaf extract. Rutin, quinic acid, and hesperidin were found only in *A. strictispinis* extracts, while caffeic acid, piceid, and fisetin were found only in *A. macrocephalus* roots. In addition, astragalin was determined only in *A. macrocephalus* subsp. *finitimus* leaves and acacetin only in *A. strictispinis* roots. Amentoflavone and genistein were not detected in any of the ethanolic extracts. In the ethyl acetate extracts, *A. macrocephalus* subsp. *finitimus* leaves were richer in terms of the analyzed compounds than its roots and *A. strictispinis* flowers. Also, in the ethyl acetate extracts, isoquercetin was found at a higher level (above 0.3 mg/g dw extract) than other phenolic compounds. In the case of the dichloromethane extracts of *A. macrocephalus* leaves and *A. strictipinis* flowers, naringenin, luteolin, apigenin, fisetin, and genistein were detected in both extracts, and among them, the content of apigenin was highest in *A. strictipinis* flower extracts. Moreover, acacetin was detected only in *A. strictipinis* flowers.

In our *Astragalus* samples, apigenin was among the abundant compounds identified in the ethanol, dichloromethane, and ethyl acetate extracts of this plant. The results of this research are in agreement with the literature data. Aydogan et al. [21] found that flavonoids, lignans, and steroidal glycosides were the main compounds in the ethanol extract of *A. strictispinus* roots from Afyonkarahisar province of Türkiye, while cycloastragenol, cycloasalgenin, 17-epicycloasalgenin, and cycloastragenol-6-O-β-D-glucopyranoside were dominant in their methanol extracts [21]. In another study, a methanol extract of *A. macrocephalus* subsp. *finitimus* was found to be rich in apigenin, hyperoside, ferulic acids, and *p*-coumaric [25]. In addition, previous studies showed that isoquercitrin has also been isolated from some other *Astragalus* species, such as *A. asper*, *A. maximus*, and *A. corniculatus* [26].

Isoquercitrin is one of the glycosidic forms of quercetin, the natural flavonol, that has a higher bioavailability than quercetin and has antioxidant and anticancer activities. Likewise, it can be effective in treating allergic reactions, diabetes, and cardiovascular diseases. Fisetin is a flavonol that has antioxidant, anti-aging, anti-inflammatory, neuroprotective, antitumor, and chemotherapeutic effects and also has potential as a senotherapeutic agent. Due to its properties, there is a desire to purify this substance and use it for the development of pharmaceuticals and food products [27]. Rutin is a flavonoid that has antioxidant, anticancer, neuroprotective, cardioprotective, etc., effects [28]. This compound was identified in the extracts of other *Astragalus* species, such as *A. glycyphyllos*, *A. cicer*, *A. campylosema*, and *A. hirsutus* [29,30]. Salicylic acid or aspirin is a well-known and important phenolic compound that has antioxidant and anti-inflammatory properties [31]. It is an important endogenous phytohormone that plays a role in the accumulation of isoflavonoids such as calycosin and calycosin-7-O-β-D-glucoside in *A. membranaceous* under chilling stress [32]. According to the results of a study, compounds such as acacetin, apigenin, and genistein were also identified in *A. membranaceous* [33]. Acacetin is a natural flavonoid that has a lot of medicinal properties, such as antitumor, antioxidant, anti-inflammatory, and cardioprotective effects. Its potential as a promising candidate for a cardiovascular drug has been raised [34]. Genistein is a natural isoflavone and a phytoestrogen that has noteworthy pharmaceutical properties, and it is used in traditional medicine as a relief for postmenopausal problems, in addition to its anticancer as well as tyrosinase and topoisomerase inhibitory activities. It reduces osteoporosis and the risk of cardiovascular attacks [35]. Amentoflavone is a natural bioflavonoid that has anti-inflammatory, antioxidant, antifungal, antidiabetic, anti-arthritis, neuroprotective, and radioprotective properties [36]. Another study reported that the main compound in the methanol extract of *A. schizopterus* is quinic acid, and rutin and hesperidin are common between *A. schizopterus*, *A. leporinus* var. *hirsutus*, and *A. distinctissimus* species [37], while hesperidin was only detected in the ethanol extract of *A. strictispinus* leaves in this study. For most plants, external factors, such as light, temperature, soil water, soil fertility, and salinity, can significantly affect some processes associated with their ability to synthesize secondary metabolites, eventually leading to changes in phytochemical profiles [38].

### 2.2. Antioxidant Potential

In the current study, two assays, e.g., DMPD and PRAP, were used to evaluate the in vitro antioxidant activity of the *Astragalus* extracts studied herein. DMPD measures the ability of antioxidants to inhibit lipid peroxidation, which is the most important type of oxidative radical damage in biological systems [39]. According to the results obtained in this study, none of the investigated extracts had antioxidant activity in the PRAP assay, while quercetin, as a positive control, was tested at three concentrations (0.1, 0.25, and 0.5 mg/mL), and their activities were 0.168 ± 0.027, 0.172 ± 0.048, and 0.834 ± 0.065, respectively. Some compounds that bind to molybdenum can inhibit the formation of the Mo(V) complex, leading to an underestimation of antioxidant activity. For example, some flavonoids, such as quercetin, rutin, catechin, etc., typically bind to metals through their hydroxyl groups. Consequently, this binding can interfere with the PRAP assay [40]. In the DMPD method, ascorbic acid, as a positive control, was tested at three concentrations (0.05, 0.1, and 0.5 mg/mL), with the results being 20.47 ± 1.42%, 38.40 ± 2.51%, and 85.86 ± 2.86%, respectively. None of the dichloromethane extracts of these species displayed antioxidant activity through the DMPD assay. The ethanol extracts of *A. macrocephalus* and the flowers of *A. strictispinus* were also inactive against the DMPD radical. Only roots (15.91 ± 2.30%) and leaves (4.89 ± 0.51%) of *A. strictispinus* had a low scavenging effect in this assay. In the case of the ethyl acetate extracts, none of the samples, except flowers of *A. strictispinus* (6.87 ± 5.01%), displayed a very low antioxidant activity in the DMPD method. These extracts might contain antioxidants that are not against the lipid peroxyl radicals involved in lipid peroxidation or antioxidants that interrupt the propagation of lipid peroxidation chains (such as vitamin E) [41]. The modest antioxidant activities observed in the present study, particularly in contrast to the high levels of individual flavonoids, such as apigenin and luteolin quantified via LC-MS, warrant a critical discussion. It is plausible that the low activity is not due to a lack of potent antioxidants, but rather a consequence of complex interactions within the crude extract. For instance, the presence of other non-antioxidant compounds (e.g., sugars, lipids, or other secondary metabolites) in the crude extract may dilute the effect of the potent antioxidants or even interact with them antagonistically. While synergistic effects between the identified phenolics are a possibility, the overall low activity suggests that any such synergy is being overshadowed by stronger antagonistic interactions. Another reason could be that the DMPD and PRAP assays may not be the ideal systems to capture the full antioxidant potential of these specific extracts.

In the literature, it was reported that DMPD assay results of *Brassica napus* L. seeds have revealed the greatest effectiveness when compared to other methods [41]. Some other researchers reported that the results of DMPD are similar to CUPRAC or ABTS assays [42,43]. In another study, the DMPD method was comparable to the ABTS method due to its very stable endpoint in results, and the inhibition power was dependent on the extract concentration [4]. In the antioxidant analysis of propolis and *Helichrysum plicatum* subsp. *pseudopliacatum*, the IC_50_ for DMPD was higher compared to the other assays [44,45]. In the case of *Cupressus sempervirens* var. *horizantalis*, the results of the antioxidant activity of the extracts exhibited differences among the applied methods. For example, the ethyl acetate extract displayed the highest activity in the DPPH radical scavenging assay, while only a small number of extracts possessed a moderate activity using the DMPD method (from 6.06 ± 0.23% to 30.34 ± 0.69%) [46].

### 2.3. Cholinesterase Inhibitory Activity

The results of the AChE and BChE inhibitory activity of the *Astragalus* extracts are presented in Table 2. The ethyl acetate extract of the leaves of *A. macrocephalus* subsp. *finitimus* exhibited the highest AChE inhibitory activity (86.27 ± 6.26%). Then, 1000 µg/mL with 20.43 ± 1.28% and 500 µg/mL with 2.19 ± 0.56% exhibited a low level of inhibition. The ethanol extract of *A. macrocephalus* possessed AChE inhibition for the leaves with 43.29 ± 2.78% and the roots with 9.76 ± 0.55%, while its dichloromethane extracts of the leaves had a moderate AChE inhibitory activity with 36.37 ± 3.17%. In most cases of *A. strictispinus,* extracts did not have inhibition against AChE, and only the dichloromethane extract of the flowers presented a very low level of AChE inhibition (below 6%). According to the literature, the ethanol extract of aerial parts of *A. dumanii* had AChE inhibitory activity (IC_50_: 1.47 µg/mL) [47]. In addition, different extracts of *A. gombiformis* were evaluated similarly, and among them, the ethyl acetate aerial part extract exhibited the most inhibitory activity (IC_50_: 110 µg/mL) [48]. The methanol extract of *A. glumaceus* leaves had 53.58% inhibition [49], while ethanol extracts of *A. crenatus* showed an IC_50_ value of 7.48 µg/mL [50]. Another study presented that the ethanolic extract of *A. neurocarpus* aerial parts had better AChE inhibition activity than its root water extract [51]. Guven et al. [4] evaluated the enzymatic activity for methanolic and water extracts of the aerial part of *A. alopecurus* and found an IC_50_ value of 1.99 and 2.45 μg/mL for AChE, respectively.

None of the extracts of *A. strictispinus* displayed inhibitory activity against BChE. Considering A. *macrocephalus,* only its leaf ethyl acetate and ethanol extracts exhibited a low BChE inhibition (29.59 ± 4.54% and 17.58 ± 4.08%, respectively). To the best of our knowledge, no data on the enzyme inhibitory activity of *A. macrocephalus* and *A. strictispinus* are present. Therefore, our results could provide new information on the biological activity poof for the genus *Astragalus*.

In the literature, the ethanol extract of aerial parts of *A. dumanii* had BChE inhibitory activity (IC_50_ value was 0.83 µg/mL) [47]. An in vitro study on *A. brachystachys* showed that the aerial parts had about 66% inhibition on BChE at 200 μg/mL [52], while for *A. crenatus*, BChE showed an IC_50_ value of 37.14 µg/mL [50]. The comparison of the results indicated that the enzyme inhibitory effect of the aerial parts of this genus was higher.

### 2.4. Antibacterial Activity

The antibacterial activity of different extracts of *A. macrocephalus* subsp. *finitimus* and *A. strictispinus* leaves were tested against *S. aureus* and *E. coli* using the diffusion method on a solid medium.

**Figure 1:**
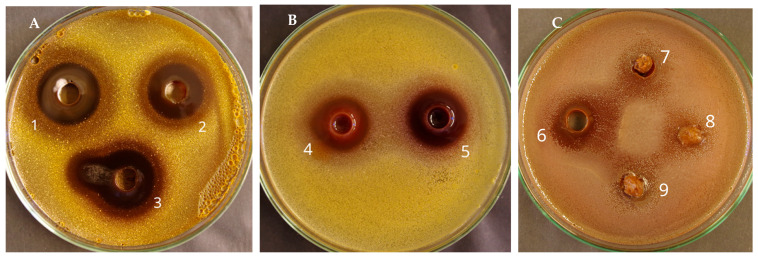
Diffusion in agar. Diameter of growth inhibition zones of the tested extracts: against *S. aureus*—panel (**A**): 1. EtOH, 2. EtOAc, 3. DCM for *A. macrocephalus*; panel (**B**): 4. EtOH, 5. EtOAc for *A. strictispinus*; against *E. coli*—panel (**C**): 6. EtOAc, 8. EtOH, and 9. DCM for *A. macrocephalus* and 7. EtOH for *A. strictispinus*.

According to the results (Table 3; Figure 1), the diameter of growth inhibition zones for *S. aureus* ranged from 13 to 25 mm, while for *E. coli*, only *A. macrocephalus* in ethyl acetate had a zone of inhibition of 13 mm. The zone of inhibition indicates antibacterial activity, and the larger the zone, the more potent the antimicrobial. *A. macrocephalus* extracts showed larger inhibition zones (over 20 mm) for *S. aureus,* characterized by higher antibacterial activity than the extracts of *A. strictispinus*. In the literature, varying degrees of antimicrobial activity in *Astragalus* species have been reported [53,54,55,56]. In contrast, some studies have reported no or low antimicrobial activity in *Astragalus* species [56]. This is likely due to the amount or type of phytochemical contents in the plant samples.

In the current study, the antimicrobial activity of the analyzed *Astragalus* extracts suggested that a high phenolic content was not always correlated with high antibacterial activity. The exhibited antibacterial activity for the tested leaf extracts could be attributed to the presence of specific phenolic compounds and the possible synergistic effects with other non-phenolic bioactive components present in the leaf extracts of *A. strictispinus* and *A. macrocephalus*.

## 3. Materials and Methods

### 3.1. Reagents and Standards

N,N-Dimethyl-p-phenylenediamine (DMPD), ascorbic acid, phosphomolybdic acid, quercetin,acetylcholinesterase (AChE), acetylcholine iodide (AChI), butyrylcholinesterase (BChE), butyrylcholine chloride (BChC), 5,5-dithio-bis-(2-nitrobenzoic) acid (DTNB), and galanthamine hydrobromide were purchased from Sigma-Aldrich (St. Louis, MO, USA).

### 3.2. Plant Materials and Extraction

**Figure 2:**
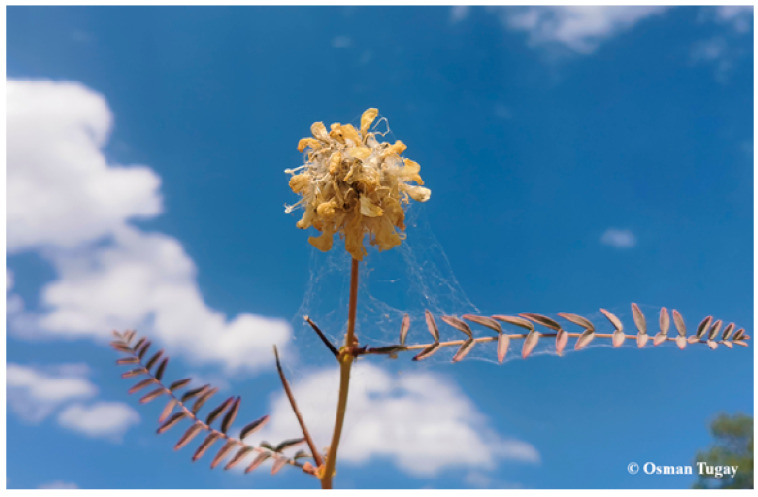
*Astragalus macrocephalus* Willd. subsp. *finitimus* (Bunge) D.F.Chamb.

**Figure 3:**
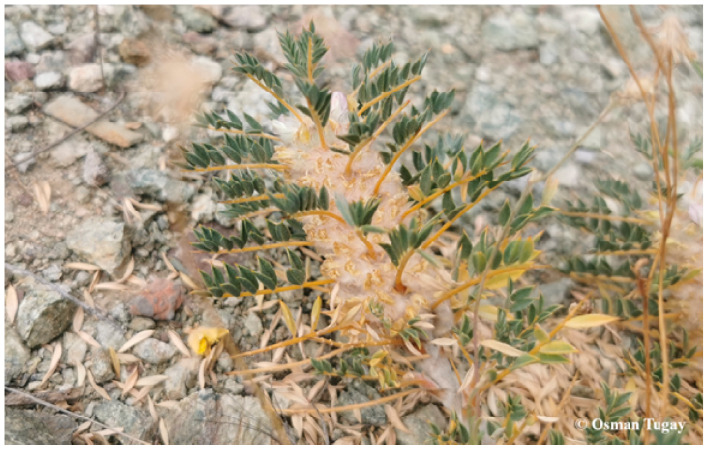
*Astragalus strictispinus* Boiss.

The plant samples of *A. macrocephalus* subsp. *finitimus* (Figure 2) and *A. strictispinis* (Figure 3) were collected from Belkuyu Village (Akören) in the vicinity of Konya province in Türkiye (37°19′38″ N, 32°25′51″ E and 37°20′58″’ N, 32°26′39″ E, respectively). The plant materials were collected in July 2024 and were identified by Prof. Dr. Osman Tugay from the Department of Pharmaceutical Botany, Faculty of Pharmacy, Selçuk University (Konya, Türkiye). Each collected plant was given the corresponding herbarium numbers as KNYA Herb. No: 30.219 for *A. macrocephalus* subsp. *finitimus* and KNYA Herb. No: 30.220 for *A. strictispinus*.

The plants were collected in the flowering season and were first divided into leaves, flowers, and roots. The plant materials were dried in shade for about 2 weeks and were powdered using a mechanical grinder. The powdered plant samples were macerated sequentially with dichloromethane, ethyl acetate, and ethanol at room temperature for 2 days. Then, the solvent parts were filtered and evaporated in vacuo to obtain the crude extracts.

### 3.3. LC-MS/MS-Based Quantitative Identification of Phenolic Compounds

The phytochemicals in different extracts of *A. macrocephalus* subsp. *finitimus* and *A. strictispinus* were assessed both qualitatively and quantitatively using a previously created and approved LC-MS/MS technique [57]. This approach was chosen since the created method may be used for a wide variety of plant species, not only the chosen species. In total, 53 phytochemical molecules, including 14 flavonoid aglycones, 13 flavonoid glycosides, 20 phenolic acids, 3 phenolic aldehydes, 1 benzopyrone, 1 stilbene glucoside, and 1 biflavonoid, were identified and measured in the species under investigation using the recently developed LC/MS/MS technique. By making up for matrix effects and analyte losses during sample preparation and analysis, internal standard solutions were used to improve the reliability of the results. The deuterated internal standards for flavonoid glycosides, flavonoids, and non-flavonoid compounds were rutin D3, quercetin D3, and ferulic acid D3, respectively. Comprehensive method validation procedures have been previously documented in the literature with respect to linearity, accuracy (recovery), limits of detection (LOD) and limits of quantification (LOQ), relative standard uncertainty (U% at 95% confidence level, k = 2), accuracy (repeatability), and precision (repeatability) between and within days [57]. Calibration curves for all 53 analytes demonstrated a strong linearity across eight concentration levels (r^2^ = 0.957–0.989), with triplicate analysis ensuring reliability. LOD and LOQ were determined through serial dilution and signal-to-noise ratio thresholds (S/N = 3:1), followed by replicate injections to confirm consistency. Precision and accuracy were assessed using intra- and inter-day analyses of spiked extracts, yielding relative standard deviations below 2.51% and recoveries ranging from 99.2% to 100.8%, confirming excellent method reliability. Additionally, relative standard uncertainties (U95) were calculated in accordance with the EURACHEM guidelines, further supporting the method’s suitability for routine phytochemical analysis. The detailed parameters related to the analytical method validation such as retention times (R.T.), molecular ions (M.I.), and fragment ions (F.I.) of the an-alytes are given in Table S1A and Table S1B of the Supporting Information File. Furthermore, the chromatograms of analyzed samples are shown in Figure S2. To ensure the robustness, reproducibility, and analytical precision of the phytochemical quantification, all experiments were conducted using three independent biological replicates.

### 3.4. Analytical Instrumentation

A triple quadrupole mass spectrometer and a Shimadzu-Nexera type ultrahigh performance liquid chromatograph (UHPLC) (Kyoto/Japan) were used to quantify 53 phenolic phytochemicals. The reversed-phase UHPLC (DGU-20A3R model) was equipped with a column oven (CTO-10ASvp type), autosampler (SIL-30AC model), binary pumps (LC-30CE model), and a degasser. The chromatographic separation was carried out using an Agilent Poroshell 120 EC-C18 model (150 mm × 2.1 mm × 2.7 m) reversed-phase analytical column. The column’s temperature was set at 40 °C. The elution gradient was composed of eluents A (water + 5 mM ammonium formate + 0.1% formic acid) and B (methanol + 5 mM ammonium formate + 0.1% formic acid). Additionally, the gradient elution profile used was 20–100% B (0–25 min), 100% B (25–35 min), and 20% B (35–45 min). Furthermore, the solvent flow rate and injection volume were set at 0.5 mL/min and 5 μL, respectively. For the mass spectrometric detection, a Shimadzu brand LCMS-8040 tandem mass spectrometer (Kyoto/Japan) equipped with an electrospray ionization (ESI) source that could be operated in both positive and negative ionization modes was used. The LC-ESI-MS/MS data was harvested and processed using Shimadzu’s LabSolutions Connect software. The multiple reaction monitoring, or MRM, approach was used to quantify the phytochemicals. The MRM technique was created to identify and measure phytochemical compounds alone, based on the screening of certain precursor phytochemical-to-fragment ion transitions. The collision energies (CE) were adjusted to achieve the maximum transmission of the intended product ions and the best possible photochemical fragmentation. The following were the MS operational settings: Drying gas (nitrogen) flow rate of 15 L/min; nebulizing gas (nitrogen) flow rate of 3 L/min; DL temperature of 250 °C; heat block temperature of 400 °C; and interface temperature of 350 °C [57].

### 3.5. Bioactivity Assays

#### 3.5.1. DMPD Radical Scavenging Assay

The principle of the assay is based on the reduction of DMPD^•+^, the purple-colored radical [58,59]. According to the method, a reagent comprising 100 mM of DMPD and 0.1 M of the acetate buffer (pH = 5.25) was freshly prepared. Then, 10 µL of the diluted samples and reference (ascorbic acid) were added to 950 µL of the mixture containing the DMPD^•+^ radical, and the absorbance was read immediately at 505 nm using a microplate reader (Molecular Devices, Spectramax ABS Plus microplate reader, San Jose, CA, USA). The experiments were performed in triplicate, and the data were computed using the formula below and presented as average values with standard deviation:

Scavenging effect (%) = [(A_1_ − A_2_)/A_1_] × 100

A_1_ = Absorbance of DMPD stock solution at 505 nm;

A_2_ = Absorbance of sample solution at 505 nm.

#### 3.5.2. PRAP Assay

The antioxidant capacity is determined by the quantification of the green color produced by the reduction of molybdenum (VI) (yellow) to molybdenum (V). The PRAP of the extracts and the reference compound (quercetin) was assessed with some modifications [60,61]. The appropriate amounts of samples were initially combined with a 10% phosphomolybdic acid solution (1000 µL) in ethanol (*w*/*v*) and incubated at 80 °C for 30 min. After incubation, a suitable mixture volume was transferred to the wells of a 96-well microplate, and the absorbance was read at the wavelength of 600 nm using a microplate reader (Molecular Devices, Spectramax ABS Plus microplate reader, USA).

### 3.6. Anticholinesterase Activity Assay

Inhibitory activity of the extracts against AChE and BChE was examined using a modified version of Ellman’s method [62,63]. In the microplate, 140 µL of buffer (pH = 8.0), 20 µL of each extract, and AChE from electric eel/BChE from horse serum were added. After 10 min of incubation, DTNB and 10 µL of the substrate (AChI for AChE and BChC for BChE) were added. In this assay, ethanol was the negative control, while galanthamine hydrobromide (100 µg/mL) was used as the positive control. The production rate and color intensity of the reaction product (2-nitro-5-thiobenzoate) were evaluated with an ELISA microplate reader (Molecular Devices, Spectramax ABS Plus microplate reader, USA) at a wavelength of 412 nm after 10 min, and the inhibitions were calculated using the following formula:

Inhibition (%) = (absorption of negative control − absorption of sample)/(absorption of negative control) × 100

### 3.7. Antibacterial Activity Assay

The antimicrobial activity studies of the analyzed *Astragalus* species were performed via an agar wall diffusion test. For this study, *S. aureus* ATCC 6538 and *E. coli* ATCC8739 strains were used according to the procedure described by Shahrivari-Baviloliaei et al. [29]. For this study, the following strains were used: Gram-positive *Staphylococcus aureus* ATCC 6538 and Gram-negative *Escherichia coli* ATCC 8739. A molten–cooled Mueller–Hinton agar (25 mL; pH = 7.5) was inoculated with 1 mL of a suspension of the appropriate bacterium at a density of 106 and then poured into the sterile Petri dish with the cylinders set. Upon solidification of the agar, the cylinders were removed to give wells with a diameter of 7 mm. Then, 0.3 mL of each extract (300 mg/mL) was added to the respective wells. Approximately 90 mg of the tested extract was placed in each well. Ampicillin (50 µg) served as a positive control. After a pre-incubation of one hour at room temperature, the plates were incubated for 24 h at 37 °C to obtain bacterial growth. After incubation, the diameter of the zone of growth inhibition was measured. After incubation, the diameter of the zone of growth inhibition was measured.

## 4. Conclusions

The present work is a comprehensive analysis of the chemical profiles and biological properties of two species of the genus *Astragalus*. To the best of our knowledge, this is the first report on the enzyme inhibitory, antibacterial activity, DMPD radical scavenging, and PRAP activity of *A. strictispinus* and *A. macrocephalus* subsp. *finitimus*. The study of the analyzed *Astragalus* species was conducted to provide the first pieces of information about their pharmacological properties. Based on LC-MS results, naringenin and apigenin were identified as two common phenolic compounds among almost all extracts from both species. In the current study, *A. macrocephalus* subsp. *finitimus* has been found to have noteworthy inhibition against cholinesterase enzymes, being AChE in particular. Among *A. macrocephalus* subsp. *finitimus* extracts, the ethyl acetate one had a more prominent activity than the other extracts, while among *A. strictispinus* extracts, the ethanol extract was more effective than the others. Moreover, all leaf extracts of the analyzed *Astragalus* species, except the dichloromethane extract of *A. strictispinus,* exhibited antibacterial activity against *S. aureus*. This study provides detailed information that may serve as the scientific basis for the use of *Astragalus* species as sources of bioactive compounds with multiple functions in the nutraceutical, cosmetic, and pharmaceutical industries. In addition, optimizing different extraction methods and conditions is also crucial for evaluating the maximum bioactivity potential of these plants. Therefore, more studies focusing on *Astragalus* species are needed to better understand their chemical profile and biological activity.

## Figures and Tables

**Table 1:** The results of phytochemical composition (mg/g dry weight) of *A. strictispinis* and *A. macrocephalus* subsp. *finitimus* extracts.

#	Compounds	EtOH				EtOAc			DCM	
		A.M. Leaves	A.M. Roots	A.S. Leaves	A.S. Roots	A.M. Leaves	A.M. Roots	A.S. Flowers	A.M. Leaves	A.S. Flowers
1	Quinic acid	nd	nd	0.75	nd	nd	nd	nd	nd	nd
6	Protocatechuic acid	0.12	0.044	0.081	nd	0.069	nd	nd	nd	nd
17	Caffeic acid	nd	0.075	nd	nd	0.013	0.044	nd	nd	nd
23	Piceid	nd	0.026	nd	nd	nd	nd	nd	nd	nd
29	Salicylic acid	0.012	nd	0.139	nd	nd	nd	nd	nd	nd
30	Cyranoside	0.211	nd	0.039	nd	0.081	nd	nd	nd	nd
33	Rutin	nd	nd	1.101	nd	nd	nd	nd	0.026	nd
34	Isoquercitrin	1.025	nd	0.365	nd	0.368	nd	nd	nd	nd
35	Hesperidin	nd	nd	0.547	nd	nd	nd	nd	nd	nd
40	Cosmosiin	0.174	0.015	0.025	nd	0.03	nd	nd	nd	nd
42	Astragalin	0.206	nd	nd	nd	0.135	nd	nd	nd	nd
44	Fisetin	nd	0.032	nd	nd	0.024	nd	nd	0.059	0.003
48	Naringenin	0.019	0.019	0.015	nd	0.018	0.008	0.008	0.009	0.045
50	Luteolin	0.1	0.302	0.037	nd	0.213	0.153	nd	0.006	0.005
51	Genistein	nd	nd	nd	nd	nd	nd	nd	0.007	0.013
53	Apigenin	0.095	0.106	0.062	nd	0.186	0.064	0.009	0.025	0.289
54	Amentoflavone	nd	nd	nd	nd	0.003	nd	nd	nd	nd
56	Acacetin	0.003	nd	0.013	0.003	nd	nd	nd	nd	0.384

A.S.: *A. strictispinus*; A.M.: *A. macrocephalus* subsp. *Finitimus*; DCM: Dichloromethane; EtOH: Ethanol; EtOAc: Ethyl acetate; nd: not detected.

**Table 2:** The results of inhibition of AChE and BChE at different extracts of *Astragalus* species and galantamine as a positive control.

Extract Type	Plant Parts	Inhibition % ± S.D. at 200 μg/mL *	
		AChE	BChE
EtOH	A.M. leaves	43.29 ± 2.78	17.58 ± 4.08
	A.M. roots	9.76 ± 0.55	-
	A.S. flowers	-	-
	A.S. leaves	-	-
	A.S. roots	-	-
EtOAc	A.M. leaves	86.27 ± 6.26	29.59 ± 4.54
	A.M. roots	25.51 ± 1.56	-
	A.S. flowers	-	-
	A.S. leaves	-	-
	A.S. roots	-	-
DCM	A.M. leaves	36.37 ± 3.17	-
	A.M. roots	-	-
	A.S. flowers	5.99 ± 0.29	-
	A.S. leaves	-	-
	A.S. roots	-	-
	Galanthamine hydrobromide (at 100 μg/mL *)	88.67 ± 2.43	69.88 ± 1.93

A.S.: *A. strictispinus*; A.M.: *A. macrocephalus* subsp. *finitimus*, DCM: Dichloromethane; EtOH: ethanol; EtOAc: ethyl acetate, S.D.: standard deviation, -: No inhibition, * Final concentration

**Table 3:** Antibacterial activity of the leaf extracts of *A. strictispinus* and *A. macrocephalus* with *ampicillin* as the positive control.

Solvent	Samples	*S. aureus* ATCC6538	*E. coli* ATCC8739
		Diameters of Zone of Inhibition (mm)	
EtOH	*A. macrocephalus*	25	0
	*A. strictispinus*	13	0
EtOAc	*A. macrocephalus*	24	13
	*A. strictispinus*	16	0
DCM	*A. macrocephalus*	24	0
	*A. strictispinus*	0	0
	*Ampicilin*	35	24

## Data Availability

The original contributions presented in this study are included in the article/Supplementary Material. Further inquiries can be directed to the corresponding authors.

## Supplementary Materials

The following supporting information can be downloaded at https://www.mdpi.com/article/10.3390/plants14223485/s1. Table S1A: Analytical method validation parameters that belong to the LC-MS/MS method. Table S1B: Analytical method validation parameters that belong to the LC-MS/MS method (Continued). Figure S1: The structural formulas of determined compounds present in Astragalus species. Figure S2: The chromatograms of analyzed samples

## Abbreviations

The following abbreviations are used in this manuscript:

ABTS	2,2′-Azino-bis(3-ethylbenzothiazoline-6-sulfonic acid
AChE	Acetylcholinesterase
AChI	Acetylcholin iodide
AD	Alzheimer’s disease
BChC	Butyrylcholine chloride
BChE	Butyrylcholinesterase
CE	Collision energy
CUPRAC	Cupric-reducing antioxidant capacity
DMPD	*N*,*N*-Dimethyl-*p*-phenylenediamine
DTNB	5,5′-Dithio-bis (2-nitrobenzoic) acid
DPPH	2,2-Diphenyl-1-picrylhydrazyl
ESI	Electrospray ionization
FRAP	Ferric-reducing antioxidant power
iNOS	Inducible nitric oxide synthase
LOD	Limits of detection
LOQ	Limits of quantification
PRAP	Phosphomolybdenum-reducing antioxidant power
UHPLC	Ultrahigh performance liquid chromatograph
