# Supplementary figures and images for: Chemical Profile and Evaluation of the Antioxidant, Anti-Enzymatic, and Antibacterial Activity of Astragalus strictispinus and Astragalus macrocephalus subsp. finitimus
Source: Plants (Basel). 2025 Nov 15;14(22):3485. doi: 10.3390/plants14223485 (PMC12655969; doi:10.3390/plants14223485)

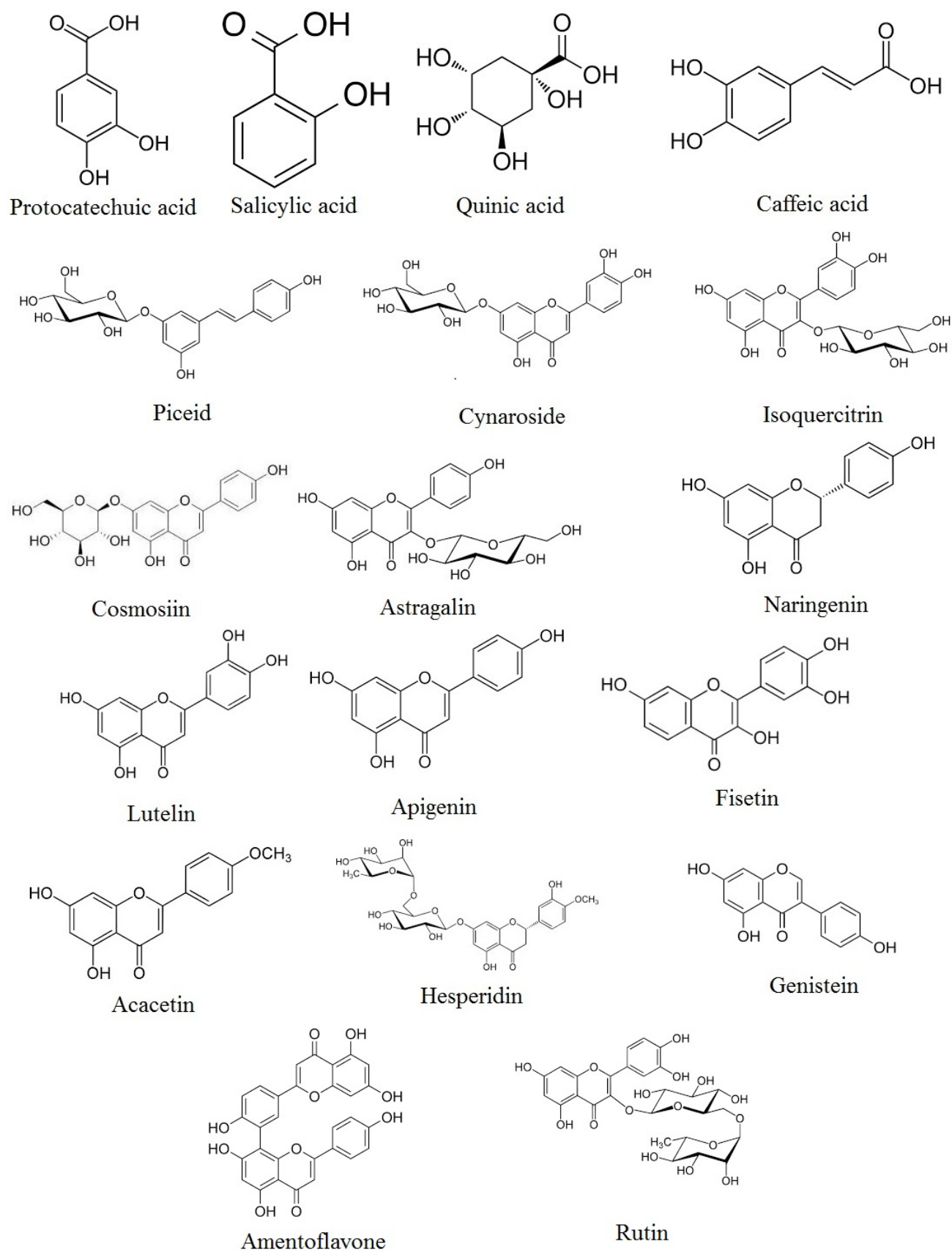

**Figure S1.** The structural formulas of determined compounds present in *Astragalus* species.

Supplement: Supplementary file 1 [file plants-14-03485-s001.zip › plants-3908989-figure S1.pdf]

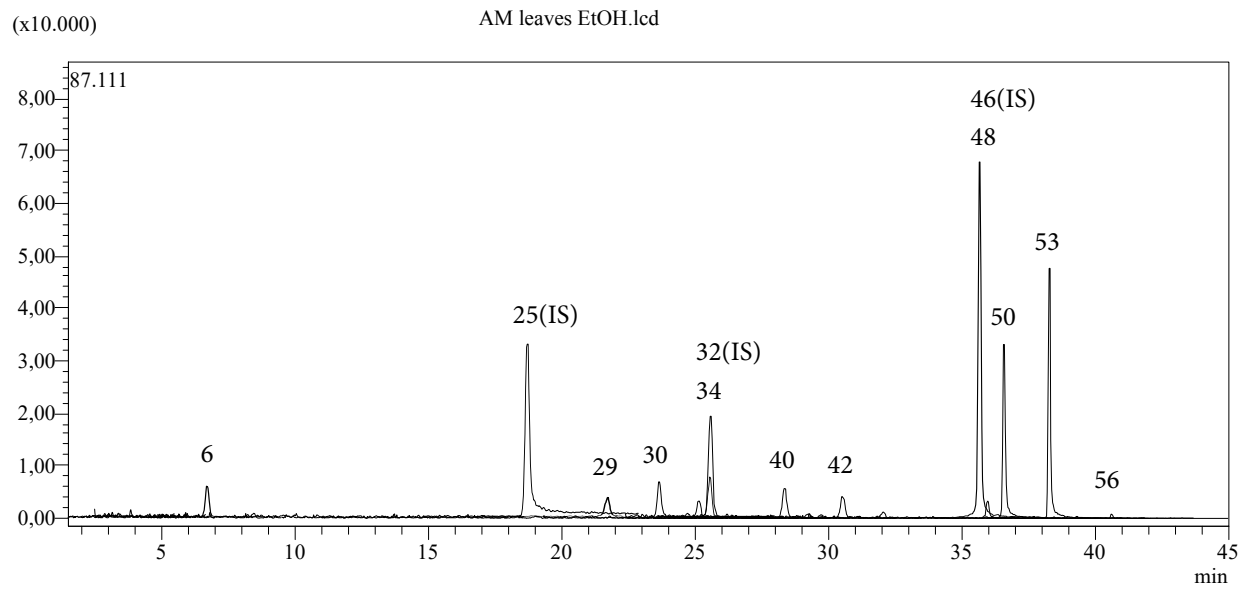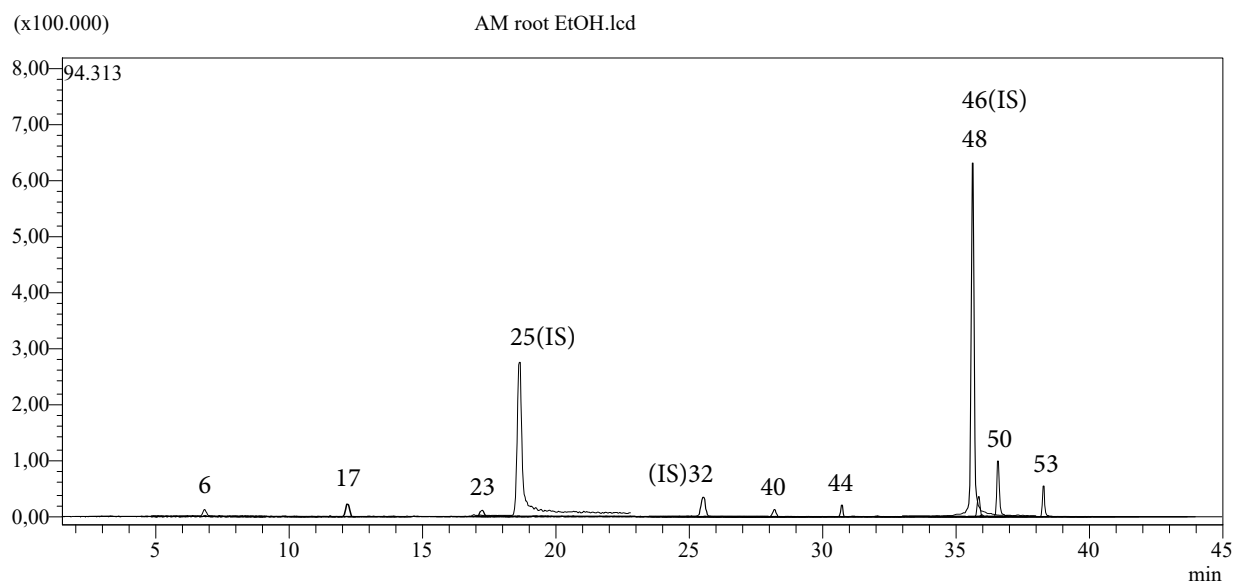

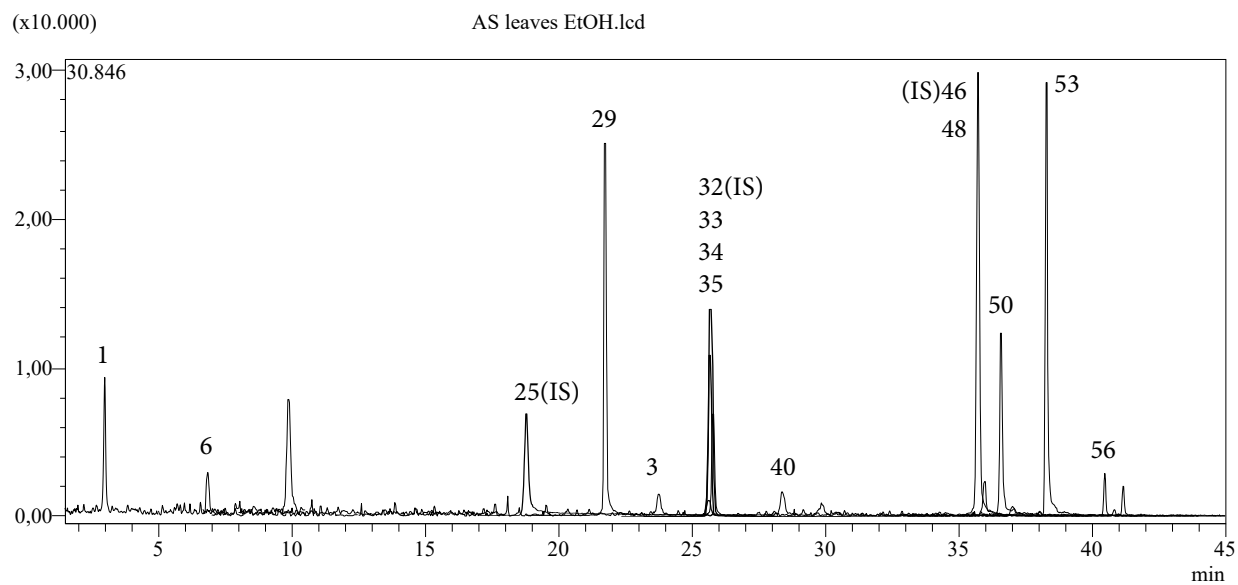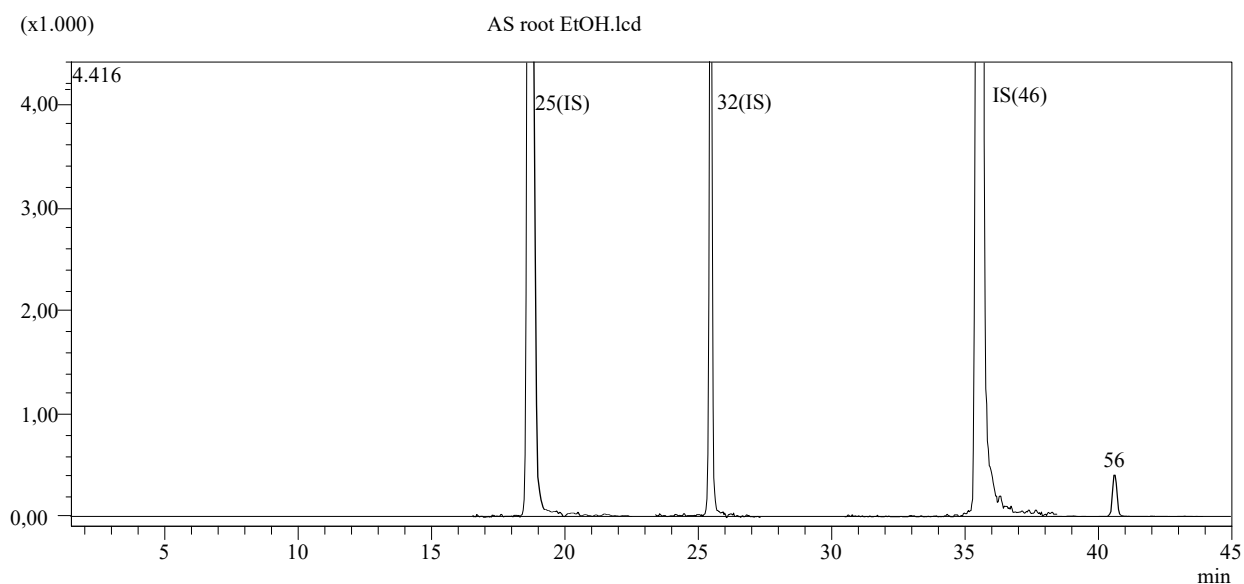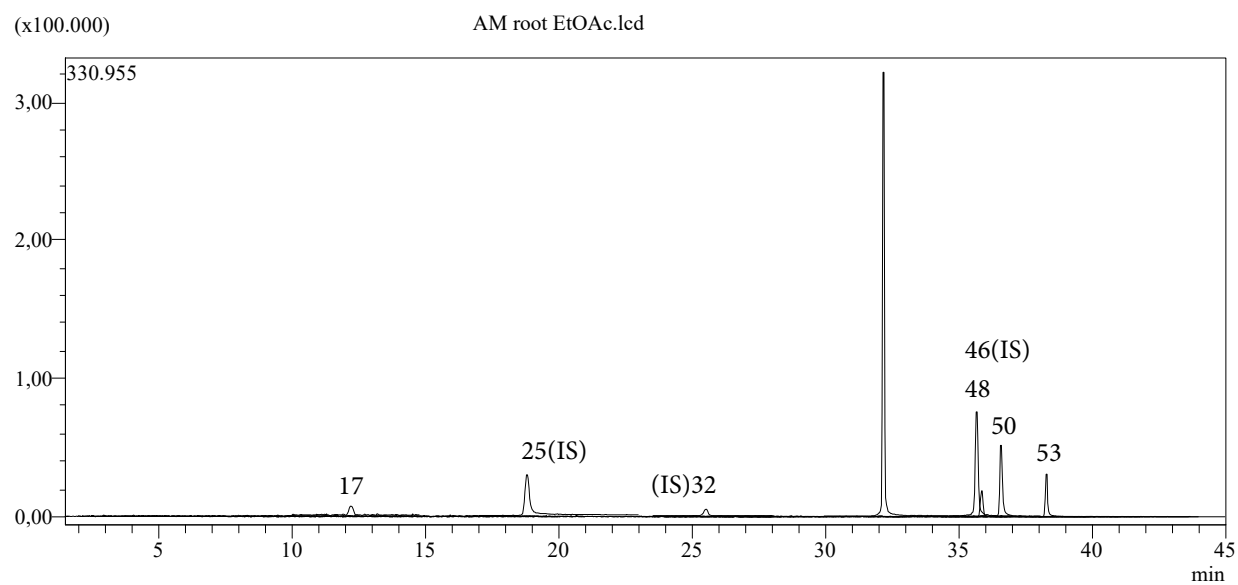

(x100.000)

AM leaves EtOAc.lcd

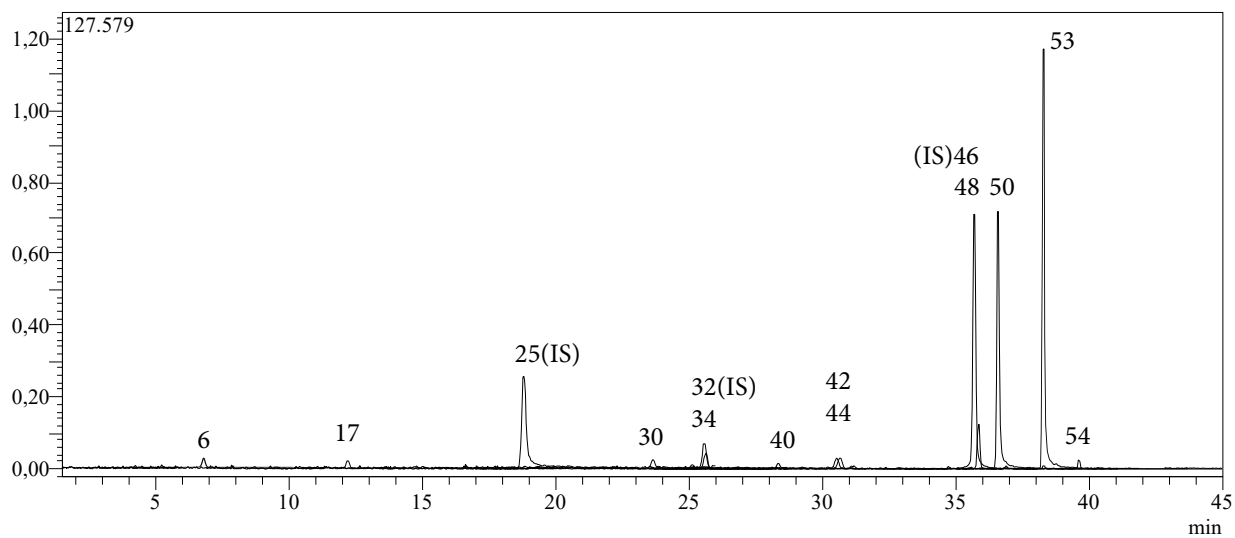

(x1.000)

AS flower EtOAc.lcd

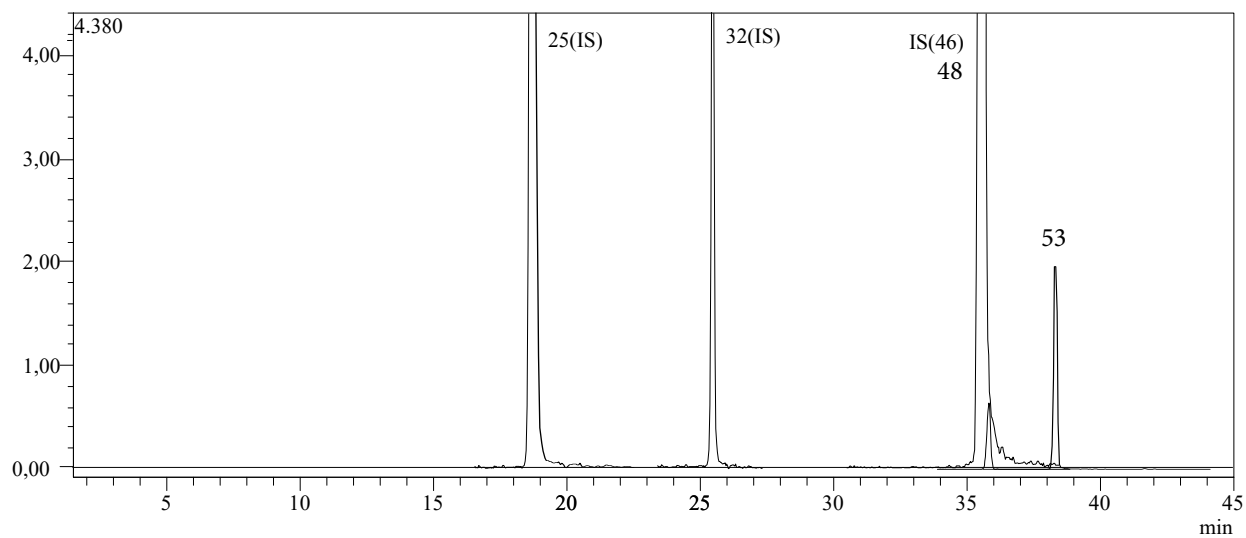

(x100.000)

AS flower DCM.lcd

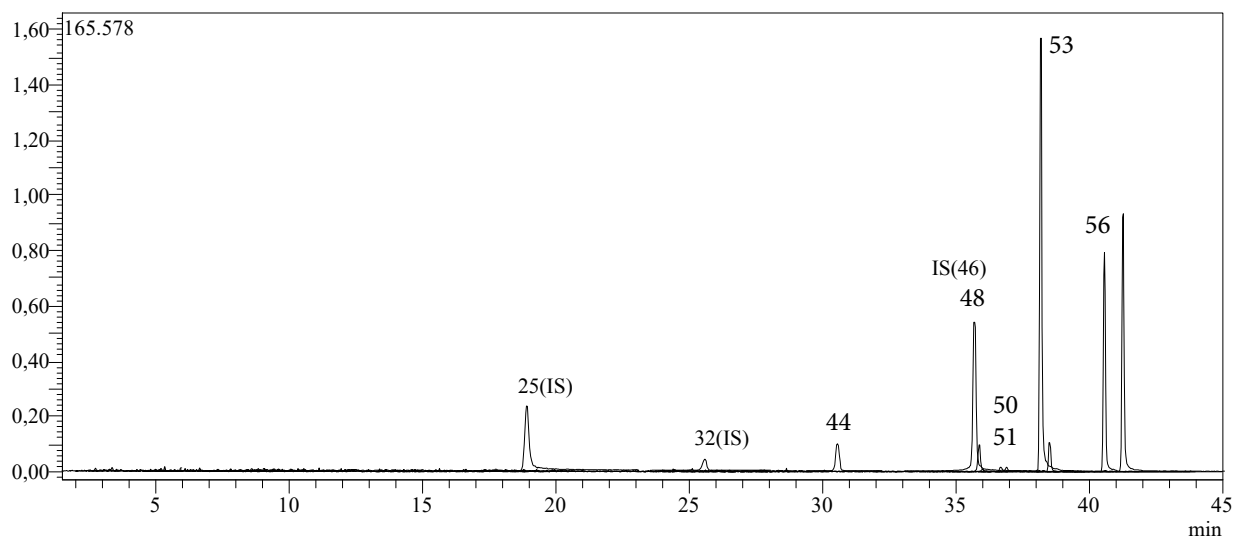

(x10.000)

AM leaves DCM.lcd

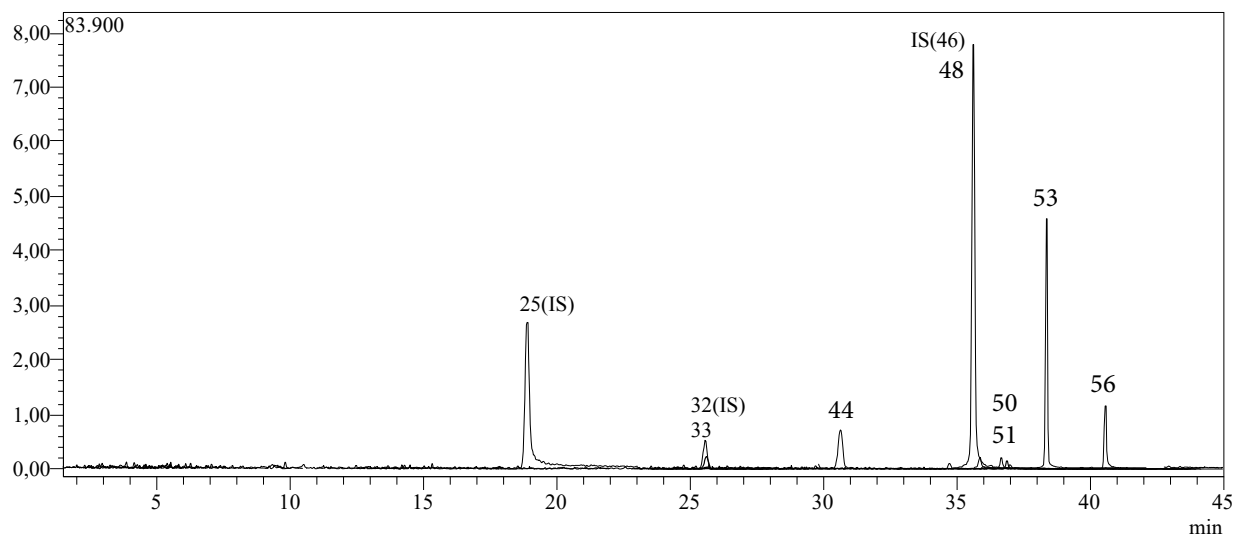

Supplement: Supplementary file 1 [file plants-14-03485-s001.zip › plants-3908989-Figure S2.pdf]
